# Application of Bayesian networks to identify factors influencing acceptability of HIV pre-exposure prophylaxis in Guilin, China

**DOI:** 10.1038/s41598-022-24965-1

**Published:** 2022-11-29

**Authors:** Lingmi Zhou, Wuxiang Shi, Sawitri Assanangkornchai, Panupong Vichitkunakorn, Jie Tang

**Affiliations:** 1 Department of AIDS Control and Prevention, Guilin Center for Disease Control and Prevention, Guilin, 541000 Guangxi China; 2 Department of Epidemiology, Faculty of Medicine, Prince of Songkla University, Hat Yai, 90110 Songkhla Thailand; 3 Health Management Unit, Faculty of Humanities and Management, Guilin Medical University, Guilin, 541004 Guangxi China; 4 Division of Computational Science, Faculty of Science, Prince of Songkla University, Hat Yai, 90110 Songkhla Thailand

**Keywords:** HIV infections, Disease prevention, Preventive medicine

## Abstract

Pre-exposure prophylaxis (PrEP) is an effective strategy to prevent uninfected individuals from contracting human immunodeficiency virus (HIV), however it must be acceptable to stakeholders in order to be effective. This study aimed to assess the acceptability of PrEP and related influencing factors. A cross-sectional survey was conducted among female sex workers (FSW), people who inject drugs (PWID), and men who have sex with men (MSM) using respondent driven sampling. Factors influencing PrEP acceptability were estimated using ordinal logistic regression and Bayesian networks. The survey included 765 eligible participants. The mean score of the perceived acceptability index was 3.9 (SD = 1.97). Multivariable logistic regression analysis revealed a higher acceptance of PrEP was associated with elder age, having other medical insurance, higher perceived utility of PrEP in facilitating prevention of HIV, higher perceived ease of use, higher perceived risk of increased risk behavior, higher perceived privacy problem in using PrEP, higher perceived comparative advantage over condom use, higher perceived comparative advantage of having sex when the urge arises, and higher perceived image of PrEP user as having sexual risky behavior, as public-minded and as health-conscious. The Bayesian network model showed perceived ease of use, perceived image of user as health-conscious, and perceived comparative advantage of having sex when the urge arises were directly associated with acceptability of PrEP. If these three factors were at a high level, 74.6% of the participants would have a high level of acceptability of PrEP. Effective education strategies to promote the acceptance of PrEP are needed. Implementation strategies should incorporate more inclusive messaging and build positive publicity for PrEP to reduce the stigma that PrEP use indicates risky behavior.

## Introduction

In 2021, approximately 1.5 million people globally were newly infected with human immunodeficiency virus (HIV). More than half of new infections are among key populations and their sexual partners, for instance, gay men and other men who have sex with men, people who inject drugs, sex workers, clients of sex workers and sex partners^1^. Female sex workers (FSW), people who inject drugs (PWID), and men who have sex with men (MSM) act as a bridge in HIV transmission in China^2–4^. An effective strategy to reduce the prevalence of HIV among them would be beneficial to the prevention of HIV. Pre-exposure prophylaxis (PrEP) is the use of antiretroviral medications on a regular basis to prevent uninfected individuals from contracting HIV^5^. After reviewing the effectiveness and safety of PrEP from clinical trial data, WHO published guidance on PrEP administration in 2012, in which PrEP is recommended to key populations^6^. In 2020, the first version of the expert consensus on PrEP for HIV^7^ in China was published and indicated that more studies were needed to better understand how this measure can be carried out in combination with other HIV-prevention strategies grounded in the local context.

Despite post-exposure prophylaxis (PEP) having had a long history in preventing HIV acquisition, PrEP is still needed for the prevention of HIV. A study showed that people receiving PEP often engage in repeated HIV-risk behaviors, with HIV diagnosis rate as high as 8.5% among MSM within a year of using PEP^8^. As recommended by the US Centre for Disease Control and Prevention, transitioning from PEP to PrEP without interruption at the completion of the 28-day PEP^9^ was associated with a reduction in HIV seroconversion rates among PEP users^10^. Some guidelines for PrEP use such as in the USA and Canada suggest that patients who seek PEP more than once should consider PrEP use after confirmation of their HIV-negative status^11,12^.

Like any other preventive strategies, PrEP has to be acceptable to people at risk for HIV to be effective^13^. Thus, it is critical to identify future users’ needs and perceptions. Based on previous studies^14–18^, main factors to determine the acceptability of PrEP can be grouped into four categories. First, individual factors such as age, education, marital status and ethnicity. Second, economic factors such as income and insurance status. Third, PrEP-specific factors such as side-effects, effectiveness and cost. Fourth, HIV-related risk factors such as HIV literacy, perceived risk of HIV, sexual risk behaviours, number of partners, and history of sexually transmitted infections (STIs). However, since PrEP is still new in most parts of China, especially in underdeveloped areas, before implementing a strategy, it is necessary to take into account the potential usage from the perspective of what is “perceived” by individuals; dimensions such as “perceived utility” and “perceived ease of use” should be considered^19^. Many acceptance models have been developed to estimate the users’ perceived acceptability. Caine et al.^20^ surveyed acceptance research over 50 years and identified the most important acceptance criteria. Since few studies have investigated acceptance criterion factors and correlations of acceptability of PrEP, especially in the underdeveloped part, it is necessary to fill this gap. In this survey, based on these criteria, we aimed to identify the attributes of PrEP that influence the acceptability, and modify them during the future implementation process to enhance key populations’ acceptance of PrEP.

Guilin is an underdeveloped city with a high burden of HIV. It is located in the northeast of Guangxi, which has the second-highest number of people living with HIV in China^21^. The main measures to prevent HIV include promoting condom use, free HIV testing, and antiretroviral therapy (ART). However, the HIV epidemic continues to spread unabated. Sentinel surveillance in Guilin revealed that the HIV positive rates among MSM, PWID and FSWs were 2.45%, 1.74% and 0.50%, respectively, which were higher than rates in the general population (0.1%)^22^. New effective supplemental preventative approaches are required.

Most previous studies used logistic regression to explore the influencing factors of acceptability based on the assumption that variables were independent of each other^14,18,23^. The complex network connections between variables were seldomly explored. Compared to logistic regression, Bayesian networks can be used to not only detect correlations but also to investigate their interrelationships^24^. Bayesian Networks, also called Bayesian Belief Networks and Belief Networks, employ directed acyclic graphs (DAG) where nodes represent variables and directed arcs imply their direct probabilistic dependences^25^. Given their interpretable and inferencing properties, Bayesian Networks have been applied widely in the medical field. For instance, they have been used to explore the correlative factors of hyperlipidemia^24^, pressure ulcers^26^ and Coronavirus Disease 2019 vaccines^27^. Hence, the objectives of this study were to explore the acceptability of PrEP and related influencing factors in order to understand how these factors affect the acceptability of PrEP. People engaging in practices contributing to the spread of HIV such as MSM, PWID, and FSWs, are one of the hard-to-reach populations^28^. To recruit this population, the Respondent Driven Sampling (RDS) technique was used. The information obtained from this study could be used to aid in the implementation of prevention strategy in China and other countries that plan to or have already implemented PrEP promotion policies.

## Results

### Participant characteristics

In total, 765 participants who met the inclusion criteria were enrolled in the survey, of which 286 were FSWs, 260 PWID and 219 MSM, respectively. The demographic characteristics of all participants are presented in Table 1. The median age of the participants was 42 years (range 18–75). Most participants were aged over 35 years (FSW: 76.4%, PWID: 91.5% and MSM: 36.7%, respectively). The majority completed junior high school (42.8%), with over half being employed. A higher proportion of MSM (71.4%) had senior high school and above education than FSWs (13.7%) and PWID (22.0%). Over half of PWID (55.8%) earned less than 1500 yuan per month, but over 80% of FSWs and MSM had a personal income 1500 yuan or more per month (98.5% and 80.7%). Most participants had urban or rural residence basic medical insurance (82.8%).

**Table 1:** Demographic characteristics of people at risk for HIV.

	Level of acceptability of PrEP			Total (N = 765) Weighted % (95% CI)
	Low (n = 314)	Medium (n = 258)	High (n = 193)	
	Weighted % (95% CI)	Weighted % (95% CI)	Weighted % (95% CI)	
**Population**				
FSW	50.4 (41.0, 58.8)	35.5 (25.9, 45.1)	38.2 (27.9, 48.5)	42.3 (33.6, 51.0)
PWID	24.5 (17.1, 31.8)	44.3 (34.7, 53.8)	34.2 (23.4, 45.3)	33.6 (25.3, 41.9)
MSM	25.2 (17.2, 33.1)	20.2 (13.8, 26.7)	27.5 (17.3, 37.8)	24.2 (17.2, 31.1)
**Age (years)**				
≤ 25	11.8 (6.2, 17.4)	6.4 (2.0, 10.9)	6.9 (2.9, 11.0)	8.8 (5.2, 12.3)
25–35	19.9 (13.5, 26.4)	18.3 (11.9, 24.8)	19.3 (10.5, 28.3)	19.2 (14.8, 23.7)
> 35	68.3 (60.6, 76.0)	75.3 (67.9, 82.7)	73.8 (64.3, 82.1)	72.0 (66.5, 77.5)
**Gender**				
Female	54.9 (46.3, 63.4)	41.7 (32.6, 50.9)	44.5 (33.7, 55.2)	47.8 (39.9, 55.8)
Male	45.1 (36.6 53.7)	58.3 (49.1, 67.4)	55.5 (44.8, 66.3)	52.2 (44.2, 60.1)
**Education**				
Primary school and below	32.7 (25.0, 40.4)	22.4 (15.4, 29.3)	23.6 (15.0, 32.2)	26.9 (21.9, 32.0)
Junior high school	38.3 (30.8, 45.8)	50.0 (42.0, 58.1)	40.7 (31.3, 50.2)	42.8 (37.5, 48.2)
Senior high school and above	29.0 (21.5, 36.6)	27.6 (20.2, 35.0)	35.7 (24.9, 46.5)	30.2 (24.8, 35.6)
**Employment status**				
Employed	65.2 (57.5, 73.0)	50.7 (41.9, 59.7)	54.8 (44.3, 65.4)	57.7 (51.3, 64.1)
Unemployed	34.8 (27.0, 42.5)	49.3 (40.3, 58.2)	45.2 (34.6, 55.7)	42.3 (35.9, 48.7)
**Income**				
< 1500 yuan per month	19.2 (12.6, 25.9)	29.2 (21.3, 36.9)	25.6 (17.4, 34.0)	24.2 (19.3, 29.1)
≥ 1500 yuan per month	80.8 (74.1, 87.4)	70.8 (63.1, 78.7)	74.4 (66.0, 82.6)	75.8 (70.9, 80.7)
**Insurance**				
None	6.7 (3.0, 10.4)	9.7 (4.6, 14.8)	11.5 (3.5, 19.5)	8.9 (5.7, 12.1)
Other	2.0 (0.3, 3.6)	1.2 (0.1, 2.4)	2.9 (0.6, 5.3)	2.0 (0.9, 3.0)
UEBMI	5.1 (2.5, 7.6)	5.6 (0.8, 10.4)	9.5 (2.4, 16.4)	6.3 (3.6, 9.1)
URRBMI	86.3 (81.5, 91.0)	83.5 (76.7, 90.3)	76.0 (66.1, 86.2)	82.8 (78.6, 86.9)

*PrEP* Pre-exposure prophylaxis, *UEBMI* Urban employee basic medical insurance, *URRBMI* Urban or rural residence basic medical insurance. *FSW* Female sex workers, *PWID* People who inject drugs, *MSM* Men who have sex with men.

### Distribution of attributes linked to PrEP acceptability

The mean score of the perceived acceptability index was 3.9 (SD = 1.97), with 41.2% (95% CI 36.0, 46.4), 33.5% (95% CI 28.5, 38.5) and 25.3% (95% CI 20.8, 29.8) showing low, moderate and high levels of PrEP acceptability, respectively. More than half of the participants perceived PrEP would facilitate the prevention of HIV and made them concerned of their health status (50.2% and 51.6%, respectively). However, most showed a low level of agreement that daily oral PrEP use was easy (34.2%). Perceived increasing risk behaviour, side-effects and privacy were at medium levels. Most had a low level of agreement on the perceived comparative advantage of PrEP. Finally, the majority of respondents had medium agreement to statements that a person who used PrEP was a public-minded person (48.2%) and health-conscious (44.3%) (Table 2).

**Table 2:** Distribution of criteria linked to PrEP acceptability among people at risk for HIV.

		Level of acceptability of PrEP			Total (N = 765) Weighted % (95% CI)
		Low (n = 314)	Moderate (n = 258)	High (n = 193)	
		Weighted% (95% CI)	Weighted% (95% CI)	Weighted% (95% CI)	
Perceived utility	**PU1: PrEP will facilitate the prevention of HIV**				
	Low	18.5 (12.9, 24.1)	4.5 (1.9, 7.0)	6.0 (1.1, 10.8)	10.6 (7.7, 13.5)
	Moderate	39.3 (31.9, 46.7)	59.0 (51.0, 67.0)	18.1 (10.2, 26.1)	40.5 (35.2, 46.0)
	High	42.2 (34.7, 49.7)	36.6 (28.9, 44.2)	75.9 (67.0, 84.8)	48.8 (43.7, 54.1)
	**PU2: using PrEP will remind me aware of my health**				
	Low	13.7 (8.7, 18.7)	8.1(3.6, 12.7)	5.1 (0.8, 9.4)	9.7 (6.6, 12.7)
	Moderate	53.0 (45.4, 60.8)	54.4 (46.4, 62.4)	16.8 (9.8, 23.7)	44.4 (39.3, 9.4)
	High	33.4 (26.0, 40.4)	37.5 (29.8, 45.2)	78.1 (70.5, 85.7)	46.0 (40.7, 51.3)
Perceived ease of use	**PEOU: daily oral taking PrEP is easy to persist**				
	Low	53.6 (46.5, 60.8)	25.3 (18.2, 32.4)	9.6 (4.7, 14.5)	33.0 (28.0, 38.0)
	Moderate	31.6 (25.2, 38.0)	52.0 (43.4, 60.5)	16.7 (9.8, 23.7)	34.7 (30.0, 39.4)
	High	14.8 (9.7, 19.8)	22.7 (16.1, 29.4)	73.7 (65.6, 81.7)	32.3 (27.2, 37.5)
Perceived risk	**PR1: PrEP will increase the risk behaviour**				
	Low	38.5 (30.9, 46.2)	30.2 (22.0, 38.4)	39.2 (29.4, 49.1)	35.9 (30.9, 40.9)
	Moderate	43.2 (35.3, 51.2)	57.3 (48.4, 66.1)	17.8 (11.2, 24.3)	41.5 (36.2, 46.8)
	High	18.2 (12.2, 24.3)	12.6 (7.3, 17.9)	43.0 (32.6, 53.5)	22.6 (18.4, 26.7)
	**PR2: PrEP will cause side-effects**				
	Low	8.4 (4.9, 12.1)	8.8 (5.1, 12.6)	21.2 (12.4, 30.0)	11.8 (8.6, 15.0)
	Moderate	47.5 (40.3, 54.8)	59.7 (51.5, 67.8)	39.0 (29.1, 49.0)	49.5 (44.1, 54.8)
	High	44.0 (36.7, 51.3)	31.5 (24.2, 38.9)	39.8 (29.6, 49.9)	38.7 (33.7, 43.8)
Perceived privacy	**PP: PrEP will cause privacy problem**				
	Low	19.6 (14.4, 25.0)	22.0 (14.7, 29.4)	27.6 (19.2, 36.1)	22.5 (18.0, 27.0)
	Moderate	52.4 (44.6, 60.1)	60.8 (52.5, 69.2)	36.8 (27.2, 46.3)	51.3 (46.0, 56.5)
	High	27.9 (21.0, 34.9)	17.2 (11.3, 23.0)	35.6 (26.7, 44.6)	26.3 (21.7, 30.8)
Perceived comparative advantage	**PCA1: I would rather use PrEP than a condom**				
	Low	74.7 (68.2, 81.2)	43.5 (34.9, 52.1)	37.0 (28.3, 46.0)	54.8 (49.5, 60.1)
	Moderate	20.1 (14.6, 25.6)	50.6 (42.4, 59.0)	19.4 (11.4, 27.2)	30.1 (25.2, 35.0)
	High	5.2 (1.1, 9.4)	5.8 (2.5, 9.2)	43.6 (33.4, 53.7)	15.1 (11.2, 19.0)
	**PCA2: I would rather use PrEP so I can have sex whenever I want to**				
	Low	85.2 (81.1, 89.4)	58.7 (50.8, 66.5)	51.9 (42.1, 61.8)	67.9 (62.7, 3.2)
	Moderate	13.4 (9.3, 17.5)	37.1 (29.2, 45.1)	22.3 (12.4, 32.1)	23.6 (18.9, 28.2)
	High	1.3 (0.1, 2.6)	4.2 (1.9, 6.5)	25.8 (16.8, 34.8)	8.5 (5.7, 11.3)
Perceived image	**PI1: person who uses PrEP often has sexual risky behaviour**				
	Low	44.6 (37.6, 51.6)	34.0 (26.6, 41.4)	43.1 (33.5, 52.7)	40.7 (35.6, 45.7)
	Moderate	31.8 (25.0, 38.5)	41.0 (32.3, 49.8)	22.7 (14.4, 31.0)	32.6 (27.5, 37.6)
	High	23.6 (16.9, 30.5)	25.0 (17.2, 32.7)	34.2 (2.4, 43.1)	26.8 (22.0, 31.6)
	**PI2: person who uses PrEP is public-minded**				
	Low	36.2 (28.8, 43.6)	17.4 (11.2, 23.5)	12.4 (6.8, 17.9)	23.9 (19.3, 28.5)
	Moderate	50.8 (43.7, 58.1)	62.2 (54.4, 68.0)	23.6 (15.3, 32.0)	47.7 (42. 6, 52.9)
	High	12.9 (8.2, 17.7)	20.4 (14.7, 26.3)	64.0 (54.4, 73.5)	28.4 (23.6, 33.1)
	**PI3: I would use PrEP because I am health-conscious**				
	Low	29.3 (22.5, 36.0)	7.7 (3.8, 11.6)	5.4 (1.5, 9.3)	16.0 (12.4, 19.6)
	Moderate	50.8 (43.6, 57.9)	61.5 (53.3, 69.8)	12.1 (6.4, 17.8)	44.6 (39.5, 49.7)
	High	19.9 (14.4, 25.5)	30.8 (22.7, 38.8)	82.6 (76.0, 89.0)	39.4 (34.5, 44.4)

*PrEP* pre-exposure prophylaxis, *PU1* perceived utility of PrEP in facilitating prevention of HIV, *PU2* perceived utility of PrEP in reminding health awareness, *PEOU* perceived ease of use, *PR1* perceived risk of PrEP in increased risk behavior, *PR2* perceived risk of side-effects, *PP* perceived privacy problem, *PCA1* perceived comparative advantage over condom use, *PCA2* perceived comparative advantage of having sex when the urge arises, *PI1* perceived image of user as having sexual risky behavior, *PI2* perceived image of user as public-minded, *PI3* perceived image of user as health-conscious.

### Influencing factors of acceptability of PrEP based on logistic regression

Based on the results of the univariate analyses, 17 variables were initially included in the multivariate analysis. Fourteen variables were significantly associated with higher acceptability of PrEP in the final model. Multivariable logistic regression analysis revealed a higher acceptance of PrEP was associated with elder age, having other medical insurance, higher perceived utility of PrEP in facilitating prevention of HIV (PU1), higher perceived ease of use (PEOU), higher perceived risk of PrEP in increasing risk behavior (PR1), higher perceived privacy problem in using PrEP (PP), higher perceived comparative advantage over condom use(PCA1), higher perceived comparative advantage of having sex when the urge arises (PCA2), and higher perceived image of PrEP user as having sexual risky behavior (PI1), as public-minded (PI2) and as health-conscious (PI3) (Table 3). Population group, gender and occupation were not significant so they were not included in the Bayesian network model.

**Table 3:** Ordinal logistic regression of influencing factors.

Nodes	Weighted % (95% CI)	UOR (95% CI)	P (LR test)	AOR (95% CI)	P (Wald’s test)	P (LR test)
**Age (Ref ≤ 25 years)**	8.8 (5.2, 12.3)		< 0.001			** < 0.001**
25–35	19.2 (14.8, 23.7)	1.97 (1.72, 2.25)		1.54 (1.30, 1.82)	** < 0.001**	
> 35	72.0 (66.5, 77.5)	1.88 (1.67, 2.12)		1.18 (1.03, 1.37)	**0.021**	
**Population (Ref = FSW)**	42.3 (33.6, 51.0)		< 0.001			
PWID	33.6 (25.3, 41.9)	1.72 (1.52, 1.95)		–		
MSM	24.2 (17.2, 31.1)	1.34 (1.18, 1.52)				
**Gender (Ref = Female)**	47.8 (39.9, 55.8)		< 0.001			
Male	52.2 (44.2, 60.1)	1.23 (1.12, 1.35)		–		
**Employment status (Ref = employed)**	57.7 (51.3, 64.1)		< 0.001			0.107
Unemployed	42.3 (35.9, 48.7)	1.23 (1.15, 1.31)		1.10 (0.98, 1.23)	0.107	
**Income (Ref ≤ 1500 yuan)**	24.2 (19.3, 29.1)		0.050			** < 0.001**
≥ 1500 yuan	75.8 (70.9, 80.7)	0.92 (0.86, 0.99)		0.76 (0.67, 0.85)	** < 0.001**	
**Insurance (Ref = None)**			< 0.001			** < 0.001**
Other	2.0 (0.9, 3.0)	1.09 (0.85, 1.39)		2.07 (1.54–2.79)	** < 0.001**	
UEBMI	6.3 (3.6, 9.1)	1.10 (0.94, 1.28)		0.56 (0.45–0.69)	** < 0.001**	
URRBMI	82.8 (78.6, 86.9)	0.61 (0.54, 0.68)		1.15 (1.00–1.33)	0.055	
**PU1 (Ref = Low)**	10.6 (7.7, 13.5)		< 0.001			** < 0.001**
Moderate	40.5 (35.2, 46.0)	1.98 (1.70, 2.32)		1.18 (0.99–1.42)	0.068	
High	48.8 (43.7, 54.1)	5.67 (4.86, 6.63)		1.66 (1.38–2.02)	** < 0.001**	
**PU2 (Ref = Low)**	9.7 (6.6, 12.7)		< 0.001			** < 0.001**
Moderate	44.4 (39.3, 49.4)	1.54 (1.33, 1.79)		0.63 (0.52–0.76)	** < 0.001**	
High	46.0 (40.7, 51.3)	6.13 (5.26, 7.16)		0.67 (0.54–0.83)	** < 0.001**	
**PEOU (Ref = Low)**	33.0 (28.0, 38.0)		< 0.001			** < 0.001**
Moderate	34.7 (30.0, 39.4)	2.84 (2.57, 3.14)		1.95 (1.72–2.21)	** < 0.001**	
High	32.3 (27.2, 37.5)	20.66 (18.36, 23.28)		5.32 (4.55–6.22)	** < 0.001**	
**PR1 (Ref = Low)**	35.9 (30.9, 40.9)		< 0.001			** < 0.001**
Moderate	41.5 (36.2, 46.8)	0.66 (0.61, 0.72)		0.89 (0.78–1.01)	0.068	
High	22.6 (18.4, 26.7)	2.63 (2.36, 2.94)		2.13 (1.84–2.47)	** < 0.001**	
**PR2 (Ref = Low)**	11.8 (8.6, 15.0)		< 0.001			** < 0.001**
Moderate	49.5 (44.1, 54.8)	0.38 (0.34, 0.43)		0.45 (0.38–0.53)	** < 0.001**	
High	38.7 (33.7, 43.8)	0.63 (0.55, 0.72)		0.48 (0.40–0.57)	** < 0.001**	
**PP (Ref = Low)**	22.5 (18.0, 27.0)		< 0.001			**0.007**
Moderate	51.3 (46.0, 56.5)	0.70 (0.64, 0.76)		1.21 (1.08–1.37)	**0.002**	
High	26.3 (21.7, 30.8)	1.82 (1.63, 2.04)		1.13 (0.97–1.32)	0.124	
**PCA1 (Ref = Low)**	54.8 (49.5, 60.1)		< 0.001			** < 0.001**
Moderate	30.1 (25.2, 35.0)	1.83 (1.68, 1.98)		1.57 (1.40–1.76)	** < 0.001**	
High	15.1 (11.2, 19.0)	18.65 (16.18, 21.55)		5.05 (4.23–6.02)	** < 0.001**	
**PCA2 (Ref = Low)**	67.9 (62.7, 73.2)		< 0.001			** < 0.001**
Moderate	23.6 (18.9, 28.2)	2.25 (2.07, 2.45)		2.21 (1.96–2.50)	** < 0.001**	
High	8.5 (5.7, 11.3)	16.80 (14.20, 19.96)		1.97 (1.55–2.50)	** < 0.001**	
**PI1 (Ref = Low)**	40.7 (35.6, 45.7)		0.019			** < 0.001**
Moderate	32.6 (27.5, 37.6)	1.12 (1.03, 1.22)		1.59 (1.43–1.77)	** < 0.001**	
High	26.8 (22.0, 31.6)	1.10 (1.00, 1.22)		1.22 (1.07–1.40)	**0.003**	
**PI2 (Ref = Low)**	23.9 (19.3, 28.5)		< 0.001			** < 0.001**
Moderate	47.7 (42. 6, 52.9)	2.01 (1.81, 2.24)		1.03 (0.90–1.18)	0.651	
High	28.4 (23.6, 33.1)	13.21 (11.68, 14.94)		2.71 (2.30–3.19)	** < 0.001**	
**PI3 (Ref = Low)**	16.0 (12.4, 19.6)		< 0.001			** < 0.001**
Moderate	44.6 (39.5, 49.7)	2.54 (2.24, 2.90)		2.84 (2.43–3.33)	** < 0.001**	
High	39.4 (34.5, 44.4)	19.27 (16.74, 22.21)		5.39 (4.53–6.42)	** < 0.001**	

*UOR* unadjusted odds ratio, *AOR* adjusted odds ratio, *LR test* likelihood ratio test, *FSW* female sex workers, *PWID* people who inject drugs, *MSM* men who have sex with men, *UEBMI*, Urban employee basic medical insurance, *URRBMI* Urban or rural residence basic medical insurance, *PU1* perceived utility of PrEP facilitating prevention of HIV, *PU2* perceived utility of PrEP in reminding health awareness, *PEOU* perceived ease of use, *PR1* perceived risk of PrEP in increasing risk behavior, *PR2* perceived risk of side-effects, *PP* perceived privacy problem, *PCA1* perceived comparative advantage over condom use, *PCA2* perceived comparative advantage of having sex when the urge arises, *PI1* perceived image of user as having sexual risky behavior, *PI2* perceived image of user as public-minded, *PI3* perceived image of user as health-conscious.

Significant values are in bold.

### Bayesian network analysis of acceptability of PrEP

**Figure 1:**
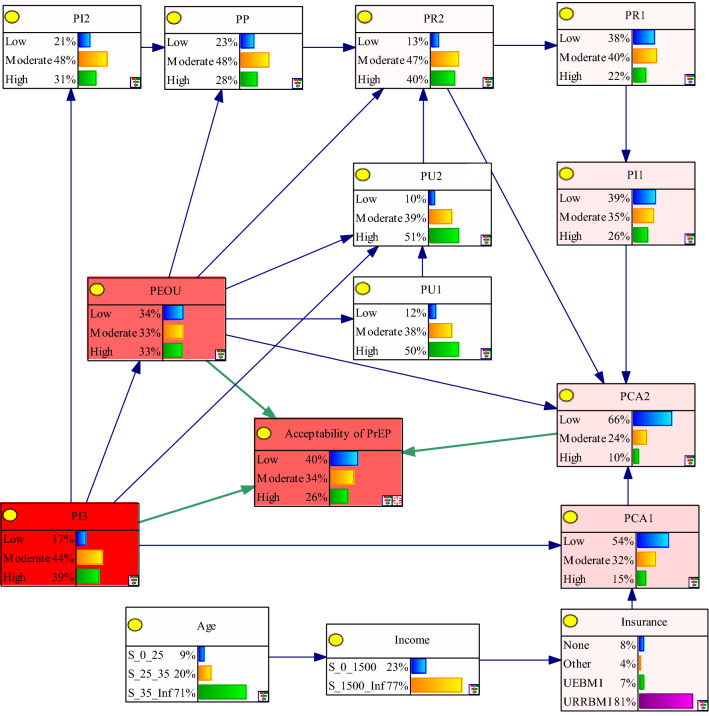
Marginal probability distribution for the Bayesian network association of acceptability of PrEP. The figure was plotted by GeNIe (3.0). *PI2* perceived image of user as public-minded, *PP* perceived privacy problem, *PR2* perceived risk of side-effects, *PR1* perceived risk of PrEP in increasing risk behavior, *PU2* perceived utility of PrEP in reminding health awareness, *PI1* perceived image of user as having sexual risky behavior, *PEOU* perceived ease of use, *PU1* perceived utility of facilitating prevention of HIV, *PCA2* perceived comparative advantage of having sex when the urge arises, *PCA1* perceived comparative advantage over condom use, *PI3* perceived image of user as health-conscious, *PrEP* Pre-exposure prophylaxis.

The overall marginal probability of selected variables was shown in Fig. 1. Perceived ease of use of PrEP (PEOU), perceived image of user as health-conscious (PI3), and perceived comparative advantage of having sex when the urge arises (PCA2) were directly associated with acceptability of PrEP. Meanwhile, the other variables were indirectly associated with acceptability of PrEP by influencing variable PCA2. Age, income and health insurance influenced the acceptability through perceived comparative advantage over condom use (PCA1) and PCA2. The other variables showed complex connections to each other and were directly or indirectly connected to PCA2.

According to the sensitivity analysis, PI3 had the highest sensitivity value (0.376), indicating that if the perception of people using PrEP being considered as health-conscious was increased to the highest level, the acceptability index would increase most in the Bayesian network. Furthermore, PEOU, PCA1 and PCA2 were also more sensitive than the other variables in impacting the acceptability level (Table 4). These four variables were thus considered as main indicators for the impact analysis.

**Table 4:** Maximum absolute sensitivity values for the acceptability of PrEP model.

Ranking	Node	Sensitivity value
1	PI3	0.376
2	PEOU	0.140
3	PCA1	0.029
4	PCA2	0.019
5	PI1	0.014
6	Insurance	0.008
7	PR1	0.007
8	PR2	0.003
9	PP	0.002
10	PU2	0.001
11	PI2	0.001
12	Income	0.001
13	PU1	0
14	Age	0

*PrEP* pre-exposure prophylaxis, *PI3* perceived image of user as health-conscious, *PEOU* perceived ease of use, *PCA1* perceived comparative advantage over condom use, *PCA2* perceived comparative advantage of having sex when the urge arises, *PI1* perceived image of user as having sexual risky behavior, *PR1* perceived risk of PrEP in  increasing risk behavior, *PR2* perceived risk of side-effects, *PP* perceived privacy problem, *PU2* perceived utility of PrEP in reminding health awareness, *PI2* perceived image of user as public-minded, *PU1* perceived utility of PrEP in facilitating prevention of HIV.

### Impact analysis

Table 5 shows the conditional probability of acceptability of PrEP when PI3, PEOU and PCA2 are in different levels. When PI3, PEOU and PCA2 were at a low level, 83.0% of participants had a low level of acceptability of PrEP. On the other hand, if all three factors were at a high level, the level of acceptability of PrEP would increase to 74.6%. However, if we kept PI3 at a moderate level when PEOU and PCA2 were at a high level, the probability that an individual would highly accept PrEP would be highest (77.8%).

**Table 5:** Conditional probability table of acceptability of PrEP.

PI3	PEOU	PCA2	Acceptability level of PrEP		
			Low	Moderate	High
Low	Low	Low	0.836	0.146	0.019
		Moderate	0.333	0.433	0.233
		High	0.167	0.167	0.667
	Moderate	Low	0.444	0.444	0.111
		Moderate	0.722	0.222	0.056
		High	0.667	0.167	0.167
	High	Low	0.697	0.061	0.242
		Moderate	0.444	0.111	0.444
		High	0.444	0.444	0.111
Moderate	Low	Low	0.690	0.276	0.034
		Moderate	0.298	0.512	0.190
		High	0.333	0.333	0.333
	Moderate	Low	0.402	0.539	0.059
		Moderate	0.375	0.577	0.049
		High	0.083	0.833	0.083
	High	Low	0.381	0.324	0.295
		Moderate	0.021	0.708	0.271
		High	0.111	0.111	0.778
High	Low	Low	0.577	0.306	0.117
		Moderate	0.194	0.528	0.278
		High	0.121	0.394	0.485
	Moderate	Low	0.363	0.274	0.363
		Moderate	0.130	0.352	0.519
		High	0.333	0.083	0.583
	High	Low	0.185	0.221	0.594
		Moderate	0.173	0.253	0.573
		High	0.079	0.175	0.746

*PrEP* pre-exposure prophylaxis, *PI3* perceived image of user as health-conscious, *PEOU* perceived ease of use, *PCA2* perceived comparative advantage of having sex when the urge arises.

## Discussion

The mean score for perceived acceptability of PrEP was at a moderate level (3.9 on a 7-point scale), with more than 40% of participants showing a low level of acceptability. Compared with a pooled estimate of 66.8% acceptability in China^29^, the acceptability in our study was lower, suggesting a need to find other ways to improve the acceptance of PrEP. Through Bayesian networks, this study illustrated the complex relationship between the acceptability of PrEP and its influencing factors. Perceived image of PrEP user as health-conscious, perceived ease of use of PrEP, and perceived comparative advantage of having sex when the urge arises had direct impacts on the acceptability of PrEP.

PrEP users often suffer from stigma. They are commonly labelled as “sexually reckless”, “promiscuous”, and “immoral”^30^. These stereotyped characteristics that form a barrier to PrEP use underscore the need to overcome this sociocultural discrimination^30,31^. However, the importance of a positive image of PrEP was seldom explored. In our study, the positive image of people using PrEP as health-conscious had a greater impact on its acceptance than the negative image of people using PrEP as frequently engaging in sexually risky behaviour. This result provides valuable clues to overcome the stigma, which is to build positive publicity for PrEP in the implementation strategies.

Perceived ease of use was an important influencing factor on the acceptability of PrEP in our study. This result was in accordance with a qualitative study that ease of use was one of the most frequently cited reasons for choosing PrEP^32^. However, most of our participants showed a moderate level of agreement about perceived ease of daily oral PrEP, which was lower than the result from a study among people who inject drugs^33^. New technology is more likely to be adopted if it is perceived to be easy to use^34^. This indicates the importance of future campaigns to promote people’s perception towards the use of daily oral PrEP.

The perceived comparative advantage of having sex when the urge arises (PCA2) was one of the positive factors for acceptability of PrEP, which may partly be because PrEP use is linked to a sense of security during sexual intercourse^35^. Additionally, a qualitative study found that using PrEP relieved sexual-related fears and shame while increasing sexual satisfaction and intimacy^36^. Previous studies exploring the sexual impact of PrEP mainly focused on risk compensation^37,38^. Our study findings, on the other hand, help to promote PrEP acceptability from a new angle such that PrEP use influences sexual lives. In addition, we found that although perceived advantage of PrEP over condom use (PCA1) did not directly connect with the outcome variable, it showed a higher sensitivity value than PCA2. This may be because PCA2 had more parent nodes, which decreased its sensitivity. Nevertheless, in our study, more than half of the participants exhibited a low level of agreement in that using PrEP has a comparative advantage while having sex. One key message to increase PrEP acceptability derived from this study is by promoting the PrEP advantage in acceding those sexually active to have sex whenever they want without the worry of contracting HIV. Most of the previous studies which explored the influencing factors of PrEP acceptability focused on individual, psychosocial and health system factors^39,40^, attributes linked to PrEP acceptability were seldom investigated. In this study we found attributes of PrEP themselves, have an important effect on acceptability of this strategy. Those findings would give clues to eliminate the stigma towards PrEP, thus facilitate the implementation of this new HIV prevention strategy.

Our findings highlight the necessity for the development of innovative strategies that can build positive publicity for PrEP, which may help reduce the stigma associated with PrEP use, increase PrEP use, and reduce the spread of HIV. It indicates that when making public campaign strategies about PrEP, policymakers should consider making more inclusive policies, such as scaling up the PrEP target populations, since if the chemoprophylaxis focuses exclusively on risk groups and specifically on stigmatized groups with a high risk of HIV, they may enhance the stigma^30^. To reduce the stigma, policies should also emphasize providing PrEP information as a health-enhancing strategy. ‘Using PrEP is taking responsibility for your own health.’ Prevention is the most effective health strategy.

Logistic regression is a widely used technique to explore epidemiological associations. However, it is significantly influenced by sample size and presupposes each variable’s independence^41,42^. Bayesian networks can reveal how influencing factors are interrelated and affect the acceptability of PrEP. This advantage helps to evaluate the multivariate internal relationship among factors, thereby provides new clues for further research. Based on the inference properties of Bayesian networks, we can predict the acceptability of PrEP on different scenarios. From the initial assessment data, we observed that PrEP was not very attractive to participants (average acceptability was 3.9 on a 7-point scale), corresponding to a moderate level. According to the Bayesian network framework, we found that helping the target people believe that using PrEP is health-conscious and can facilitate safe sex, while promoting the ease-of-use of PrEP, can largely increase the high-level acceptability from the original 25.3% to 74.6%. This gives a clue to conduct PrEP prevention strategies in underdeveloped areas.

Our study has several limitations. First, to decrease the dependence on initial seeds, normally a small number of seeds is used to ensure sufficient successive waves in the RDS process^43,44^. However, after one month, it was clear that we would not achieve the desired sample size, thus we increased the number of seeds in each participant group. Perhaps out of fear of disclosing their privacy, some participants, particularly those recruited through HIV voluntary counselling and testing clinics, refused to refer their friends to our study. Certain seeds of PWID recruited from methadone maintenance treatment clinics expressed a desire to avoid interaction with other PWID in order to avoid relapse. This may have resulted in selection bias, diminishing the study’s representativeness. However, we selected seven voluntary counselling and testing clinics and four methadone maintenance clinics that covered the people with a higher risk of HIV infection. Second, the direct arcs in our Bayesian networks do not represent cause-effect relationships; rather, they illustrate how the variables interact. Only networks formed by directed edges in a causal relationship serve as evidence of causality^45^. Third, there is no consensus regarding the variable selection for Bayesian networks, in our study we selected variables based on significance in univariate and multivariate regression, this may be misleading^46^, caution should be used when interpreting our results.

## Conclusion

By utilizing Bayesian networks, we have enabled researchers to gain a better understanding of the impact of various factors on PrEP acceptability. Our results also allow policymakers to simulate the level of acceptability in different scenarios in order to identify areas where the prevention method can be improved. PrEP was moderately accepted among people at risk for HIV in Guilin. More strategies to promote PrEP acceptability are needed. The finding that perceived positive image, perceived ease of use and perceived comparative advantage of having sex when the urge arises as the most important attributes of PrEP suggests that incorporating these characteristics into PrEP strategies may help increase its acceptability. Implementation strategies should incorporate more inclusive messaging and build positive publicity for PrEP to reduce the stigma that PrEP use indicates risky behavior.

## Methods

### Study design and setting

A cross-sectional survey was conducted in HIV voluntary counselling and testing clinics, methadone maintenance treatment clinics and non-governmental organization offices in Guilin, China.

### Participants and data collection

The target population for this study was people at risk for HIV, including female sex workers (FSW), persons who inject drugs (PWID) and men who have sex with men (MSM). An eligible participant was one who was aged 18 years or above, self-reported HIV negative or status unknown, able to give informed consent, living in Guilin for at least one year, and within the last 12 months, belonging to one of the following three groups representing the population at risk for HIV:

- FSW, defined as having commercial sex;
- PWID, defined as having injected drugs;
- MSM, defined as having oral or anal sex with men.

From November 2020 to April 2021, a respondent-driven sampling (RDS) method^47^ was employed to recruit those hard-to-reach populations. Firstly, we identified 3–4 well connected and trusted participants from each of the population groups as “seeds” to recruit eligible participants from their social networks. However, after about 1 month of recruitment commencement, the initial seeds could not refer any respondents, we therefore increased the number of seeds of each group. Altogether, 49 FSWs, 99 PWID and 55 MSM were identified as seeds. Each participant received three coupons to recruit not more than three new participants. Each new participant was given three coupons after completing the questionnaire and so on until the expected number of samples was reached. Each participant received 50 yuan ($8 USD) for their participation in the study and 20 yuan ($3 USD) for successfully recruiting each peer or partner into the study.

### Measures

A structured questionnaire was used to gather information about demographics, acceptability of PrEP and the criteria linked to PrEP acceptability.

#### Acceptability of PrEP

Given that PrEP is currently unfamiliar to the target population in Guilin, a brief introduction of PrEP was provided. We adopted the term “likely to use” to indicate the likelihood of PrEP acceptability, based on prior studies which assessed acceptability^14,48–50^. Participants were asked: “Overall, how likely would you use PrEP?” The response, which was on a 7-point Likert scale, ranged from 1 (very strongly unlikely) to 7 (very strongly likely), reflecting a willingness to use or willingness to try a product^51^. The outcome was classified into three degrees of acceptability: 1–3 (low level), 4–5 (moderate level), and 6–7 (high level)^52^.

#### Identification of influencing factors

An initial set of criteria for PrEP acceptability was first prepared based on research by Caine et al.^20^, in which they provided a framework with the ten most important factors related to product acceptance. To identify factors suitable for our model, we conducted an in-depth interview with two participants in each group (six in total) prior to data collection. Finally, six major criteria of PrEP acceptability were included in this study, including (1) perceived utility (PU), (2) perceived ease of use (PEOU), (3) perceived risk (PR), (4) perceived privacy (PP), (5) Perceived comparative advantage (PCA), and (6) perceived image (PI). The questionnaire for this section was composed of 12 statements concerning factors influencing acceptability of PrEP, each with responses on a Likert scale ranging from 1 (strongly disagree) to 7 (strongly agree). The score of each statement was then classified into three levels (low, moderate and high) by the same cut point used for acceptability of PrEP.

### Statistical analysis

To adjust for potential bias in respondent-driven sampling, we used RDS Analyst^53^ to estimate the prevalence and 95% CI of each variable. We combined data from three population groups and normalized the weights of each population group by multiplying each participant’s sampling weight by a further weight, which was equal to the mean of individual weights in each population divided by the mean weights of all three population groups^54^. Normalized sampling weight was used in ordinal logistic regression to select variables potentially associated with level of acceptability. Under univariate analysis, variables with a p-value from the likelihood ratio test of less than 0.05 were selected into initial multivariate model. Variables with a p-value < 0.05 from the multivariate analysis were then included in the Bayesian network model. Each factor’s result was reported as an odds ratio and accompanying 95% confidence interval (CI). R version 4.1.0 was used to conduct the analyses.

The Bayesian network analysis was constructed in GeNIe 3.0^55^. Each factor obtained from the final multivariate logistic regression model represents a node. Greedy Thick Thinning Algorithm was used to learn the structure. Considering the logic and accuracy, we adjusted the structure according to two principal rules: (1) demographic characteristics as the attributes of people; the other nodes cannot be the parent nodes of them, and (2) acceptability of PrEP as the consequence, which cannot be the parent nodes of the other nodes. In the parameter learning step, an expectation–maximization algorithm^56^ with uniform parameter initialization was set, the marginal probabilities estimated by the corresponding state frequencies were used to denote the conditional probability of the selected root variables, and the child nodes were illustrated by posterior probability. We performed a sensitivity analysis based on the method of Kjaerulff and van der Gaag^57^, which distinguishes the node that can affect the posterior probability of the target variable.

### Ethics statement

This study was approved by the Ethics Committee of the Faculty of Medicine, Prince of Songkla University, Thailand (REC.63-321-18-1) and Guilin Center for Disease Control and Prevention (2020#033). To protect participants’ anonymity and eliminate the risk that signatures could reveal their personal information, verbal informed consent was obtained after an introduction of the study was provided. The Ethics Committee of the Faculty of Medicine, Prince of Songkla University, Thailand (REC.63-321-18-1) and the Guilin Center for Disease Control and Prevention (2020#033) both authorized verbal informed consent.

### Informed consent statement

Participant consent was waived due to the facts that FSW, PWID, MSM are vulnerable population and signature may cause potential harm, thus verbal informed consent was obtained.

## Data Availability

Availability of data and materials the data used in this study are available from the corresponding author (S.A.) and first author (L.Z.) on reasonable request.
